# Quinoa Reduces High-Fat Diet-Induced Obesity in Mice via Potential Microbiota-Gut-Brain-Liver Interaction Mechanisms

**DOI:** 10.1128/spectrum.00329-22

**Published:** 2022-05-18

**Authors:** Ting-Ye Wang, Si-Yu Tao, Yan-Xiang Wu, Tian An, Bo-Han Lv, Jia-Xian Liu, Yu-Tong Liu, Guang-Jian Jiang

**Affiliations:** a School of Traditional Chinese Medicine, Beijing University of Chinese Medicine, Beijing, China; b Zhong Li Science and Technology Limited Company, Beijing, China; c Gansu Pure High-Land Agricultural Science and Technology Limited Company, Lanzhou, Gansu, China; Lerner Research Institute

**Keywords:** quinoa, obesity, gut microbiota, microbiota-gut-brain-liver axis

## Abstract

The gut microbiota is important in the occurrence and development of obesity. It can not only via its metabolites, but also through microbiota-gut-brain-liver interactions, directly or indirectly, influence obesity. Quinoa, known as one kind of pseudocereals and weight loss food supplements, has been high-profile for its high nutritional value and broad applications. In this context, we produced high-fat diet-induced (HFD) obese mouse models and assessed the efficacy of quinoa with saponin and quinoa without saponin on obesity. We explored the potential therapeutic mechanisms of quinoa using methods such as 16S rRNA, Western blotting, Immunohistochemical (IHC). Our results indicated that quinoa can improve the obese symptoms significantly on HFD mice, as well as aberrant glucose and lipid metabolism. Further analyses suggest that quinoa can regulate microbiota in the colon and have predominantly regulation on *Bacteroidetes*, *Actinobacteria* and *Desulfovibrio*, meanwhile can decrease the *F*/*B* ratio and the abundance of *Blautia*. Contemporaneously, quinoa can upregulate the expression of TGR5 in the colon and brain, as well as GLP-1 in the colon, liver and brain. while downregulate the expression of TLR4 in the colon and liver, as well as markers of ER stress and oxidative stress in livers and serums. Beyond this, tight junctional proteins in colons and brains are also increased in response to quinoa. Therefore, quinoa can effectively reduce obesity and may possibly exert through microbiota-gut-brain-liver interaction mechanisms.

**IMPORTANCE** Gut microbiota has been investigated extensively, as a driver of obesity as well as a therapeutic target. Studies of its mechanisms are predominantly microbiota-gut-brain axis or microbiota-gut-liver axis. Recent studies have shown that there is an important correlation between the gut-brain-liver axis and the energy balance of the body. Our research focus on microbiota-gut-brain-liver axis, as well as influences of quinoa in intestinal microbiota. We extend this study to the interaction between microbiota and brains, and the result shows obvious differences in the composition of the microbiome between the HFD group and others. These observations infer that besides the neurotransmitter and related receptors, microbiota itself may be a mediator for regulating bidirectional communication, along the gut-brain-liver axis. Taken together, these results also provide strong evidence for widening the domain of applicability of quinoa.

## INTRODUCTION

Quinoa (Chenopodium quinoa Willd.) is a plant initially from the Andean regions. Its composition are highly nutritious and has attracted many attentions from the scientific community (1). As a kind of promising functional and medicinal food with high value, quinoa has been found to be beneficial for the treatment of obesity and related endocrine diseases including type 2 diabetes. A study (2) found that inclusion of Latin American crops including quinoa reducing glycemic index could help preventing metabolic diseases.

Obesity has become increasingly prevalent worldwide not only as a major driver of non-communicable diseases but also as a disease itself (3). Gut microbiota is considered as a vital influence on obesity, a previous study has found that quinoa supplement could regulate the intestinal microbiota of obese mice and changes trend toward the lean mice (4). Previous studies have found that quinoa could regulate microbiota composition in the colon, but whether it plays a regulatory role through microbiome-gut-brain-liver axis still remains undefined.

The diet has been well established to play a key role in shaping the composition of gut microbiota which is highly variable. Consumption of high-fat diets causes dysbiosis, whether in microbial quantity or diversity. In turn, the gut microbiota plays an essential role in obesity processes (5, 6). For high-fat diet-induced (HFD) obese mice, dysbiotic gut microbiota reduces the activation of takeda G protein-coupled receptor 5 (TGR5), which leads to the decrease of biosynthesis of secondary bile acids and then causes disorders of bile acid metabolism and bacterial overgrowth and finally contributes to liver injury (7). In the meantime, the reduction of TGR5 activation may decrease the secretion of glucagon-like peptide-1 (GLP-1), which amplifies obesity (8). Lipopolysaccharide (LPS) due to microbiota dysbiosis and decreased TGR5 lead to the stimulation of toll-like receptor 4 (TLR4) inflammatory pathway. Under the combination of multiple factors, the chronic inflammatory lesions induce an increase in the intestinal permeability, which results in systemic inflammation (6). Next, transfer of the gut microbiota and its metabolites through the disrupted intestinal barrier influences various organs, such as the liver, thereby leading to ER stress of liver (9). In the meantime, gut microbiota may regulate obesity through their indirect effect on the brain via the gut flora metabolism pathway and the intestinal barrier and blood-brain barrier (BBB) (8).

In this study, we sought to reveal the modulation actions of quinoa on obesity through microbiota-gut-brain-liver axis by analyzing microbiota in the colon and try to understand the function of quinoa in ameliorate obesity.

## RESULTS

### Quinoa reduced obesity in HFD mice.

**FIG 1:**
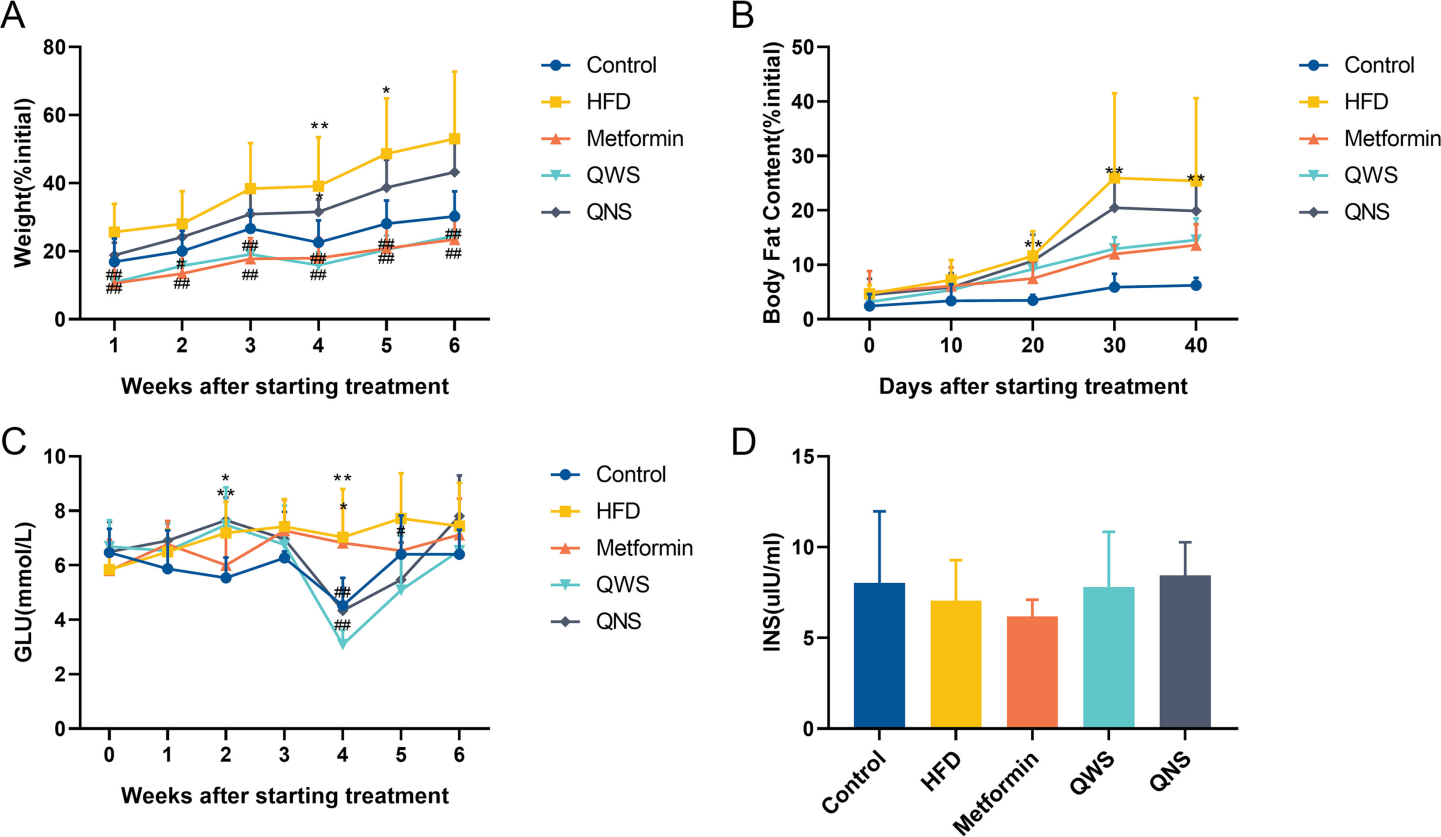
Quinoa reduced obesity in HFD mice. (A) Changes of body weight growth. (B) Changes of body fat content. (C) Changes of fasting blood glucose. (D) Changes of insulin levels. Values are expressed as the mean±SD of six mice in each group. *, *P*<0.05; **, *P*<0.01; compared with the control group and ^#^, *P*<0.05; ^##^, *P*<0.01, versus the HFD group. A repeated measurement process of general linear model was used to conduct one-way ANOVA analysis for repeated measured data (body weight, body fat and fasting blood glucose), and multivariate analysis process of variance was used to make comparisons between groups on each time point. One-way ANOVA, non-parametric test, Dunnett’s T3 were used to analyze the comparisons between groups.

During 6-week intervention period, the rate of weight gain of the HFD group was higher than the control group, and the body weight growth of the intervention groups was lower than the HFD group (Fig. 1A). The growth rate of weight in the HFD group was significantly higher than the control group in the fourth (*P*<0.01) and fifth (*P*<0.05) week of intervention, whereas the weight gain was obviously lower (*P*<0.01) in the Metformin group and the quinoa with saponin (QWS) group than the HFD group throughout the whole duration. For the growth rate of body fat content, the situation was comparable (Fig. 1B). After 20 days intervention, the HFD group presented with a higher growth rate of body fat content than the control group (*P*<0.01), which continued until the end of the trail. The growth rates of body fat content in the QWS group and the quinoa without saponin (QNS) group were lower than that in the HDF group over the same period, but the differences were not statistically significant. In addition to body weight and body fat, we also measured fasting blood glucose and insulin levels (Fig. 1C and D). The fasting blood glucose of the HFD group was higher than the control group in the fourth week after intervention (*P*<0.01), while the glucose level tended to be lower in the QWS group than the HFD group in the fifth week (*P*<0.05). The insulin level was slightly lower in the HFD group, but differences between groups were not significant. Taken together, these results suggest that quinoa could reduce the body weight, body fat and fasting blood glucose of HFD obese mice, and the QWS group performed a slight advantage in efficacy.

### Quinoa ameliorated serum lipids, inflammation, and oxidative stress.

**FIG 2:**
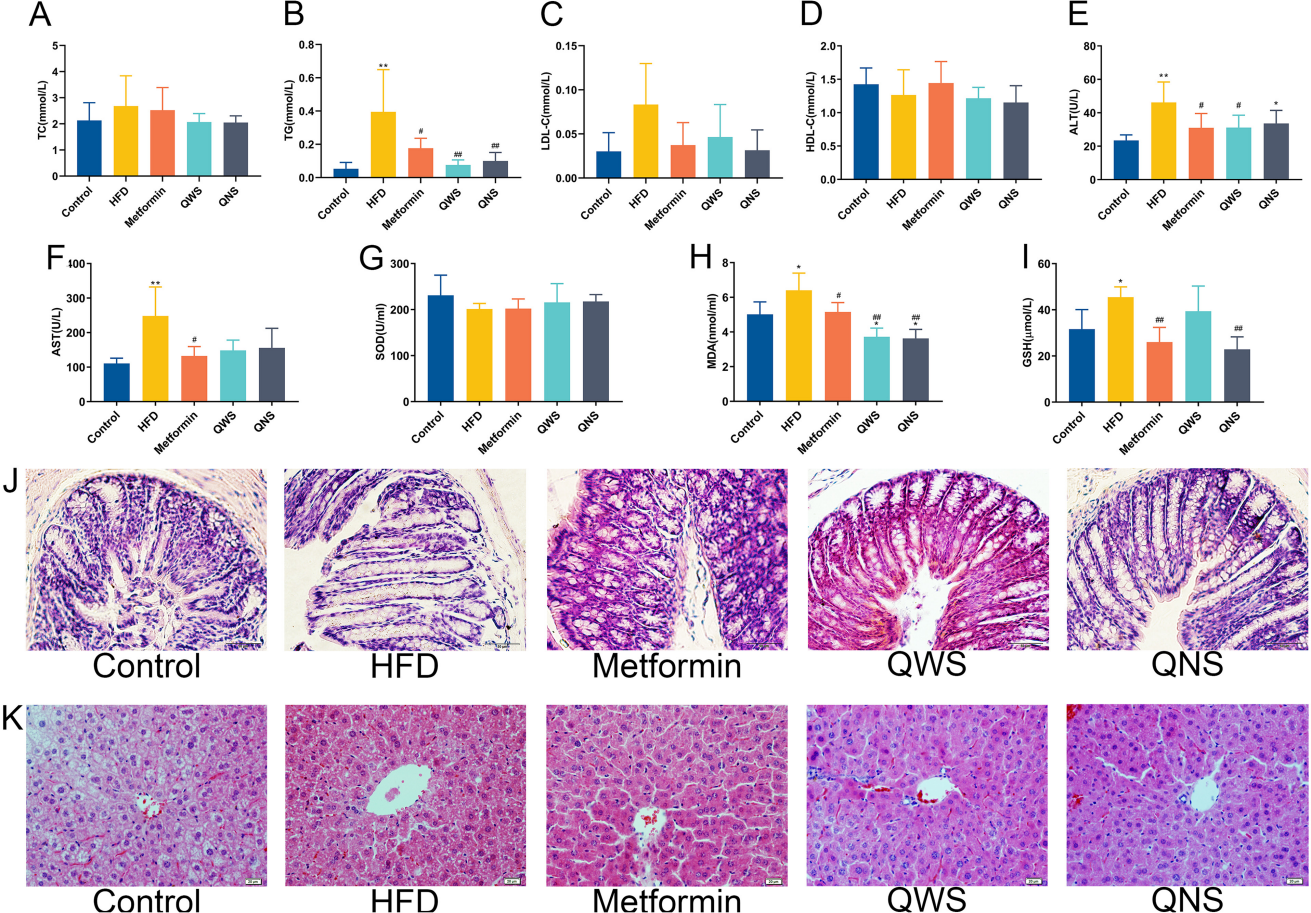
Quinoa ameliorated serum lipids, inflammation and oxidative stress. (A) Total cholesterol (TC), (B) Serum triglyceride (TG), (C) Low-density lipoprotein cholesterol (LDL-C), (D) High-density lipoprotein cholesterol (HDL-C), (E) Serum alanine aminotransferase (ALT), (F) Serum aspartate aminotransferase (AST), (G) Superoxide dismutase (SOD), (H) Malondialdehyde (MDA), (I) Glutathione (GSH). (J) HE staining of colon tissue sections. (K) HE staining of liver tissue sections. Values are expressed as the mean±SD of six mice in each group. *, *P*<0.05; **, *P*<0.01; compared with the control group and ^#^, *P*<0.05; ^##^, *P*<0.01, versus the HFD group, determined by ANOVA and non-parametric test.

Serum biomarkers were measured after 6-week intervention. Comparing to the control group, the HFD group showed significant trends of upregulation in TG, LDL-C, ALT and AST (*P*<0.01) (Fig. 2). The levels of TG were obviously decreased in the QWS group and the QNS group (*P*<0.01) compared with the HFD group. In addition, the level of LDL-C in the QNS group (*P*<0.01) and ALT in the QWS group (*P*<0.05) expressed lower versus the same indicators in the HFD group. Moreover, the results of hematoxylin-eosin (HE) staining in liver tissues (Fig. 2K) indicated that the control group had a normal structure of hepatic lobule, and the liver cells arranged orderly. Obvious liver damage (hepatic steatosis and diffuse degeneration of hepatocytes) was observed in the HFD group. After intervention, the degeneration of hepatocytes was obviously reduced in the Metformin, QWS, QNS group. The HE staining of colon tissues (Fig. 2J) showed that the lamina propria was exposed and the colonic mucosal structure was damaged in the HFD group, whereas the colon of the Metformin, QWS, QNS group was improved, with only slight epithelial structural damage.

SOD, MDA and GSH were determined to assess the degree of oxidative stress in obese mice (Fig. 2G–I). The SOD content decreased, and the MDA (*P*<0.05) and GSH (*P*<0.05) level increased in the HFD group versus the control group. SOD was only mildly affected by interventions, while MDA and GSH were significantly downregulated in the QNS group (*P*<0.01), meanwhile the level of MDA was flat between the QNS group and the QWS group (*P*<0.01). These results suggested that quinoa might improve lipid metabolism and liver function and alleviate inflammation and oxidative stress associated with obesity, and no significant differences were detected between the QWS group and the QNS group.

### Quinoa regulated the gut microbiota of HFD obese mice.

**FIG 3:**
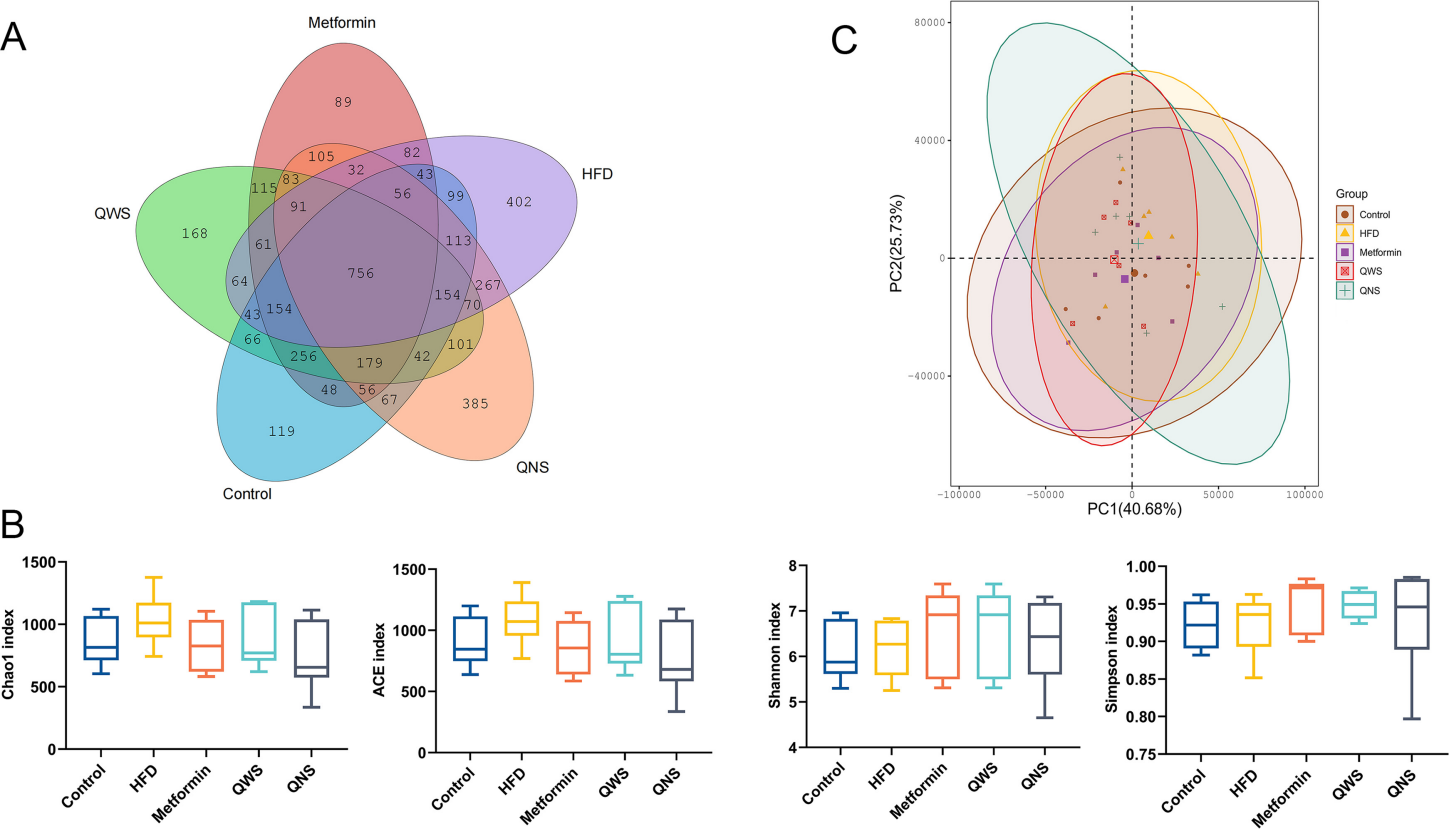
Diversity and richness of the gut microbiota and principal-component analysis. (A) Venn chart basing on the OTUs of gut microbiota. (B) Analyses of alpha-diversity (Chao1, abundance coverage-based estimator (ACE), Shannon and Simpson indices) in each group. (C) Principal component analysis (PCA) plot about the colonic microbiota.

To assess the impact of quinoa in gut microbiota, cecal contents were harvested for microbial analysis. Venn diagram analysis of OTUs at 97% sequence similarity showed the total OTU number was 4366, with 756 common OTU species in all groups, and the unique OTU species of the HFD group were the most (Fig. 3A). Alpha-diversity was calculated to evaluate the differences in microbial richness and diversity in five groups (Fig. 3B). We assessed microbial richness using the Chao1 and abundance coverage-based estimator (ACE), estimated microbial diversity using the Shannon and Simpson indices. The HFD group showed an mild increase in the richness of the gut microbiome than the control group, and the results were not statistically significant. The principal-component analysis (PCA) showed large separations of microbiota composition among the control, HFD, QNS group, and similar composition among the control, Metformin, QWS group (Fig. 3C).

**FIG 4:**
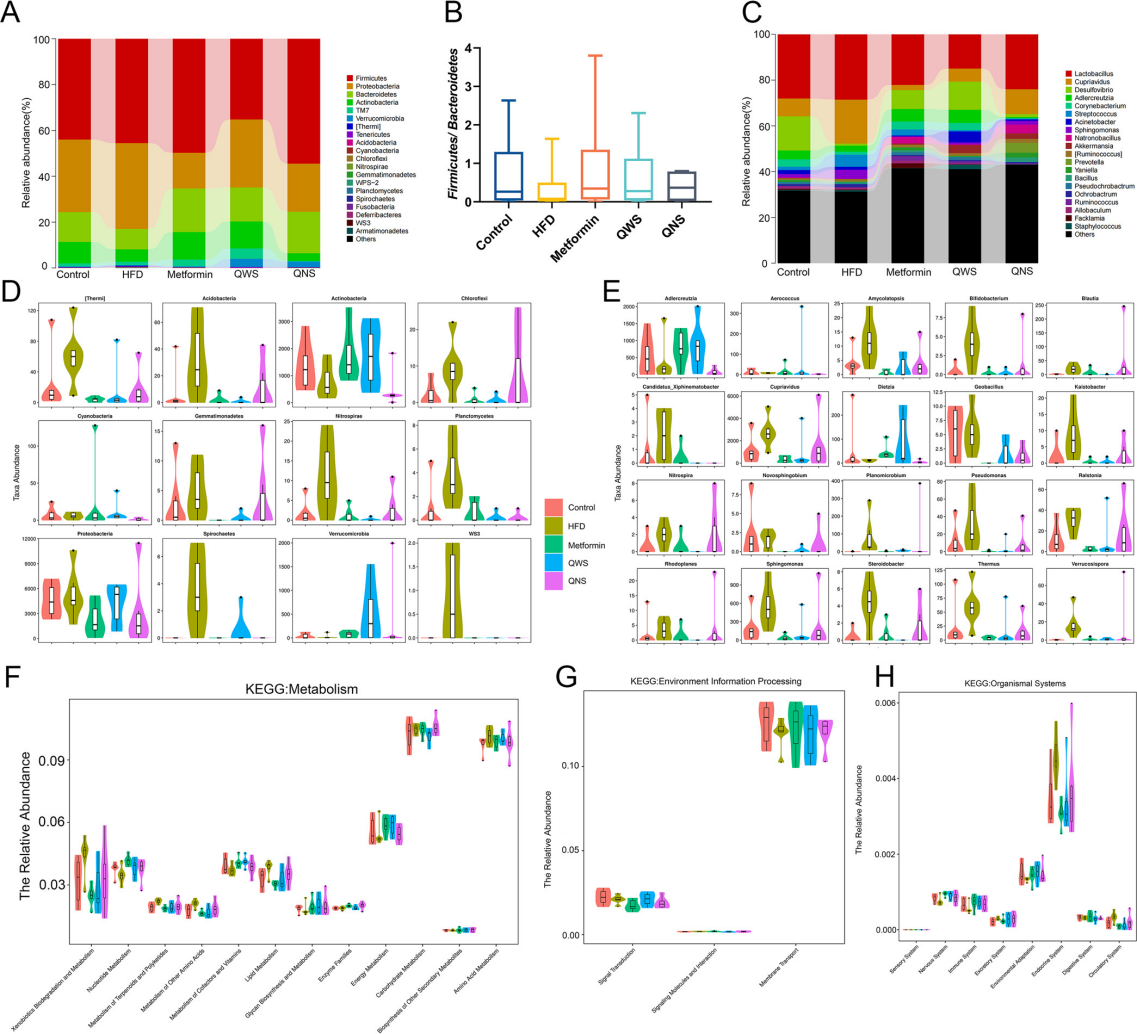
Quinoa regulated the gut microbiota composition of HFD obese mice. (A) Gut microbiota composition at the phylum level among groups. (B) The ratio of *Firmicutes*/*Bacteroidetes* (*F*/*B*) in five groups. (C) Gut microbiota composition at the genus level among groups. (D) The abundance distribution of the top 12 taxa at the phylum level with the most significant differences between groups. (E) The abundance distribution of the top 20 taxa at the genus level with the most significant differences between groups. (F to H) PICRUSt-inferred KEGG enrichment analysis.

To compare the differences in the composition of cecal contents microbial between each group, taxonomy of microbes was profiled at phylum and genus levels. At the phylum level (Fig. 4A), Firmicutes, Proteobacteria, Bacteroidetes, Actinobacteria make up a large part. The abundance of Proteobacteria was increased in the HFD group compared with the control group, while Bacteroidetes and Actinobacteria were decreased. Compared to the HFD group, Proteobacteria abundance reduced in the Metformin and QNS group, whereas Bacteroidetes and Actinobacteria increased in the Metformin and QWS group. Notably, the increased Firmicutes/Bacteroidetes abundance ratio is positively related to obesity (10). The ratio of Firmicutes/Bacteroidetes was robustly decreased in the Metformin, QWS and QNS group, which indicated quinoa with saponin and quinoa without saponin could treat obesity via influencing intestinal microbiota (Fig. 4B). At the genus level, Lactobacillus and Cupriavidus were the predominant bacterial genus among all groups (Fig. 4C). The abundance of Cupriavidus in the HFD group was higher than control group, while Desulfovibrio was lower than the control group. The QWS and QNS group downregulated the abundance of Cupriavidus and upregulated Desulfovibrio versus the HFD group.

Metastats analysis was employed to investigate bacteria with a statistically significant difference at the phylum and genus levels. The top 12 taxa at the phylum level and top 20 taxa at the genus level with the most significant differences were shown in Fig. 4D and E. The abundance of Acidobacteria, Chloroflexi, Nitrospirae, Planctomycetes, Spirochaetes, and *WS3* was higher in the HFD group at the phylum level, meanwhile the abundance of Actinobacteria was lower than other groups. Remarkably, at the genus level the abundance of Blautia which has been confirmed higher expression in obese individuals (11) was higher in the HFD group, and quinoa could reduce the abundance. Beyond this, the abundance of Amycolatopsis, Bifidobacterium, Candidatus*_*Xiphinematobacter, Cupriavidus, Kaistobacter, Pseudomonas, Sphingomonas, Steroidobacter increased greatly in the HFD group, while the abundance of Adlercreutzia was markedly lower in the HFD group than the control, Metformin, and QWS group.

To better understand the biological significance of the alterations in cecal contents microbiota composition, we performed PICRUSt based on the KEGG pathway using 16S rRNA data. Genes relevant to metabolism, environmental information processing, organismal systems were more abundant (Fig. 4F–H). In detail, the relative abundance of xenobiotics biodegradation and metabolism, metabolism of terpenoids and polyketides, lipid metabolism, amino acid metabolism, membrane transport and endocrine system was higher in the HFD group than other groups, which may be associated with obesity lesions.

### Quinoa may regulate obesity through the gut-liver axis.

**FIG 5:**
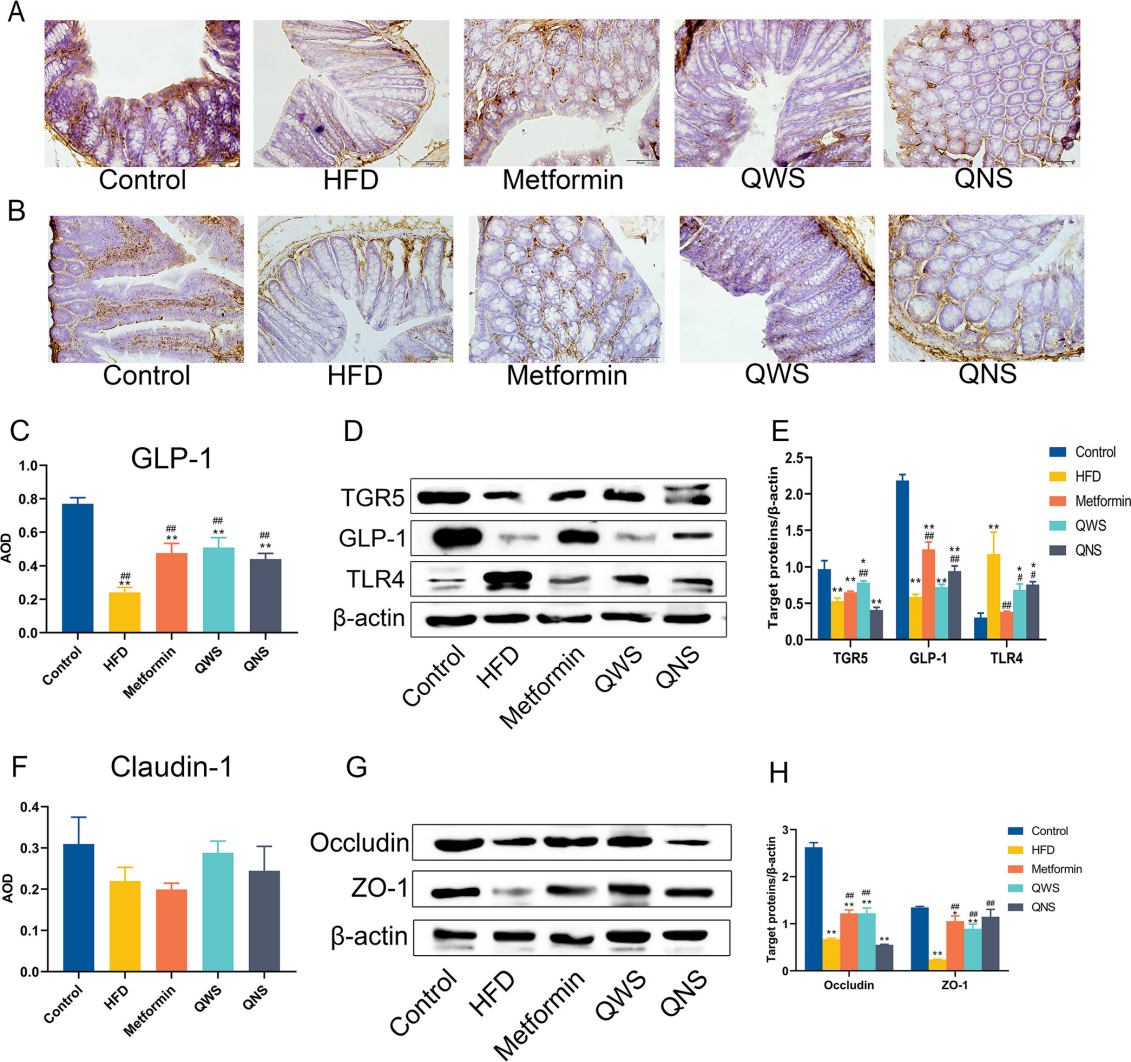
Quinoa may regulate TGR5, GLP-1, TLR4 and typical tight junctional proteins in the colon. (A) IHC of GLP-1 in the colon. (B) IHC of Claudin-1 in the colon. (C) Quantification average optical density (AOD) values of GLP-1 in the colon by Image J. (D) Representative Western blot of TGR5, GLP-1 and TLR4 in the colon. (E) Quantitative assessment of the Western blot analysis results of TGR5, GLP-1 and TLR4 by Image J. (F) Quantification AOD values of Claudin-1 in the colon by Image J. (G) Representative Western blot of occludin and ZO-1 in the colon. (H) Quantitative assessment of the Western blot analysis results of occludin and ZO-1 by Image J. *, *P*<0.05; **, *P*<0.01; compared with the control group and #, *P*<0.05; ##, *P*<0.01, versus the HFD group, determined by ANOVA and non-parametric test.

TGR5, a G protein-coupled receptor and bile acid receptor, is expressed in many organs such as liver, colon. We assessed TGR5 expression in colon tissues, and the data showed that there was a significant decrease of TGR5 in the HFD group, comparing to the control group (Fig. 5D and E). The expression of TGR5 elevated in the Metformin group, while slightly increasing trends were observed in the QWS and QNS group. Further analysis of the GLP-1 in colon revealed that in the HFD group the level of GLP-1 was lower than the control group (*P<*0.01), whereas these changes were reversed by quinoa (Fig. 5A, C–E). The results demonstrated that quinoa could induce GLP-1 secretion via TGR5 activation.

TLR4 is a crucial mediator of the inflammatory process in the colon, so we detected TLR4 expression. TLR4 in the HFD group was remarkably higher than control group, and there was a clear trend of decreasing in intervention groups (Fig. 5D and E). The dysbiosis of gut microbiota could lead to not only inflammation but also increased the permeability of the gut. Claudin-1, occludin, and ZO-1 are typical tight junctional proteins, and their decreases contribute to the disruption of the intestinal barrier function. (Fig. 5B, F–H). In the colon, occludin and ZO-1 in the HFD group were significantly decreased versus the control group. Occludin in the Metformin and QWS group significantly increased than the HFD group. ZO-1 in the Metformin, QWS and QNS group was markedly upregulated. The expression of Claudin-1 was not statistically significant between the control and the HFD group, whereas the results revealed quinoa with saponin had the effect of upregulation.

**FIG 6:**
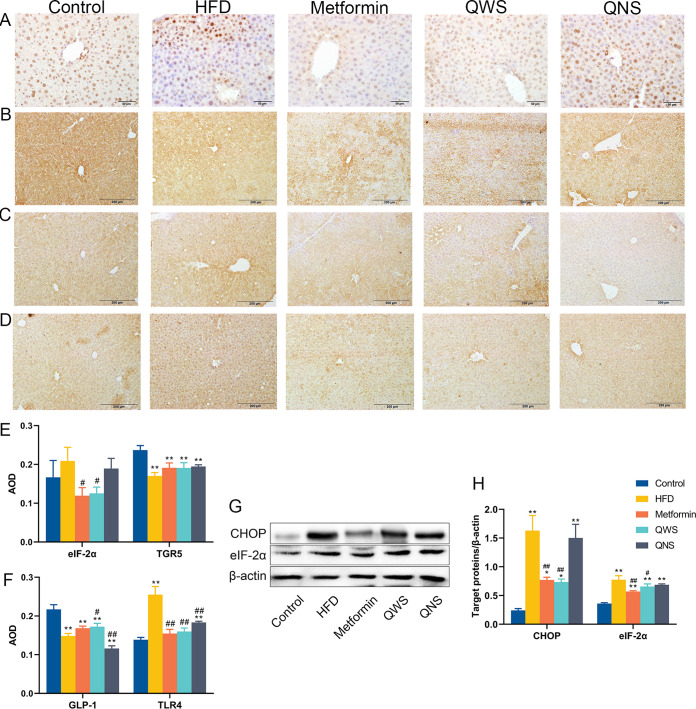
Quinoa may regulate TGR5, GLP-1, TLR4 and endoplasmic reticulum (ER) stress in the liver. (A) IHC of phosphorylated eukaryotic initiation factor 2 alpha (eIF-2α) in the liver. (B) IHC of TGR5 in the liver. (C) IHC of GLP-1 in the liver. (D) IHC of TLR4 in the liver. (E) Quantification AOD values of eIF-2α and TGR5 in the liver by Image J. (F) Quantification AOD values of GLP-1 and TLR4 in the liver by Image J. (G) Representative Western blot of the transcription factor C/EBP homologous protein (CHOP) and eIF-2α in the liver. (H) Quantitative assessment of the Western blot analysis results of CHOP and eIF-2α by Image J. *, *P*<0.05; **, *P*<0.01; compared with the control group and #, *P*<0.05; ##, *P*<0.01, versus the HFD group, determined by ANOVA and non-parametric test.

We measured expressions of the endoplasmic reticulum (ER) stress markers (GHOP and eIF-2α) in liver tissues to explore the level of liver damage (Fig. 6A, E, G, and H). The results showed that the expression of CHOP and eIF-2α were higher in the HFD group than in the control group, which indicated an ER stress response in liver tissues of obese mice and the results matched the above findings of HE staining. Quinoa with saponin reduced the expression of CHOP and eIF-2α in the liver. TGR5 and GLP-1 in the liver were significantly reduced in HFD group than the control group, and TLR4 was aberrantly elevated. Quinoa with saponin could enhance the expression of GLP-1, however TLR4 decreased in the QWS and QNS group. (Fig. 6B–F).

### Quinoa also regulated the brains of obese mice in the same pathway.

**FIG 7:**
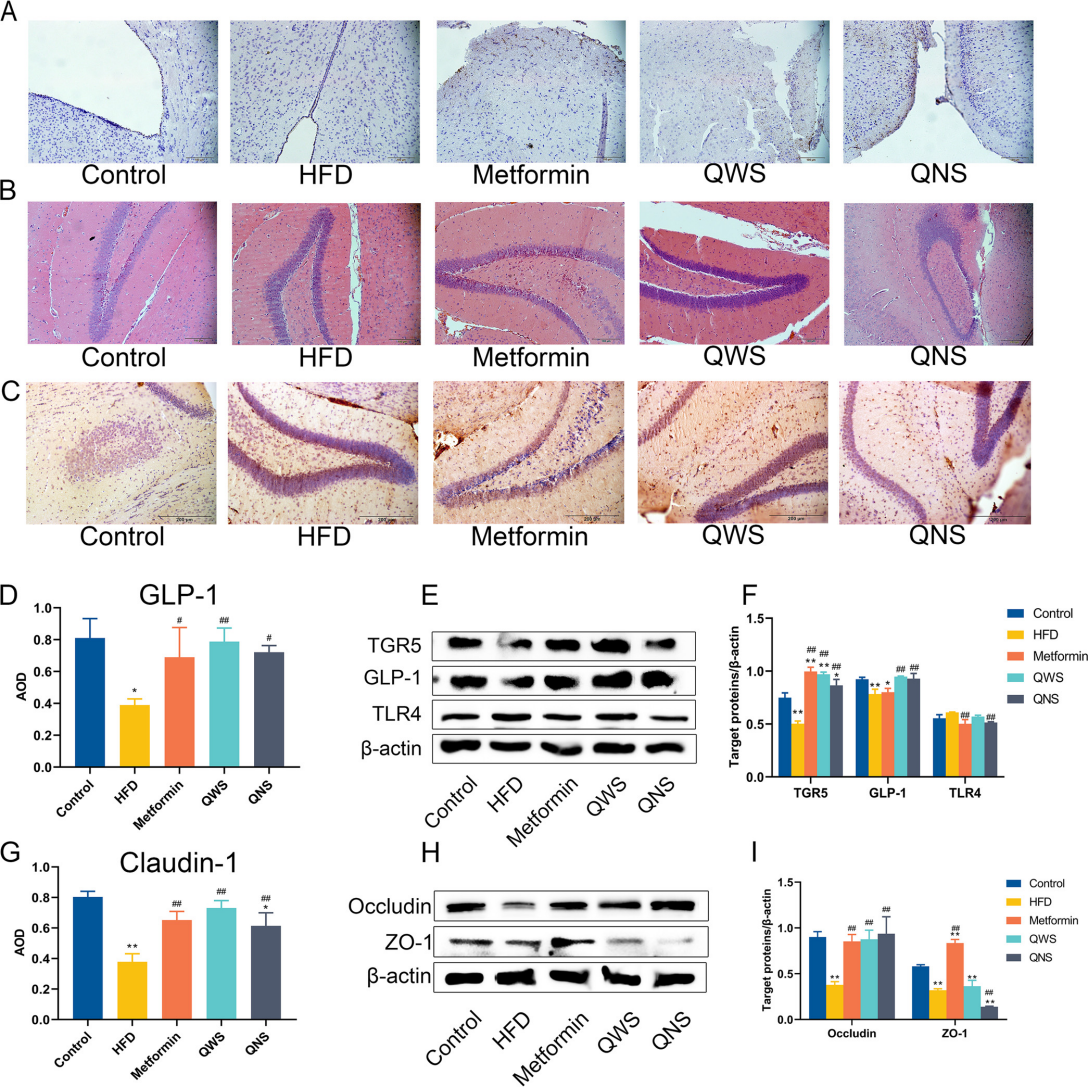
Quinoa regulated the same receptors in brains of obese mice. (A) IHC of GLP-1 in the brain. (B) HE staining of brain tissue sections. (C) IHC of Claudin-1 in the brain. (D) Quantification AOD values of GLP-1 in the brain by Image J. (E) Representative Western blot of TGR5, GLP-1 and TLR4 in the brain. (F) Quantitative assessment of the Western blot analysis results of TGR5, GLP-1 and TLR4 by Image J. (G) Quantification AOD values of Claudin-1 in the brain by Image J. (H) Representative Western blot of occludin and ZO-1 in the brain. (I) Quantitative assessment of the Western blot analysis results of occludin and ZO-1 by Image J. *, *P*<0.05; **, *P*<0.01; compared with the control group and #, *P*<0.05; ##, *P*<0.01, versus the HFD group, determined by ANOVA and non-parametric test.

TGR5 has been already confirmed to express in the brain and may participate in the anti-obesity effects (12). We examined TGR5 expression in the brain, and the Western-blot results showed that TGR5 was lowly expressed in the HFD group compared with the control group, while quinoa increased the expression of TGR5 in the brain (Fig. 7E and F). As for GLP-1, the trends in brain were similar to the trends in the colon (Fig. 7A, D and E and F). The results displayed the content of GLP-1 in brain decreased in the HFD group versus the control group. These abnormalities of GLP-1 in the HFD group can be corrected by intervention of quinoa.

The severity of inflammation was evaluated by the expression of TLR4 in brain (Fig. 7E and F) and the morphological changes of HE staining (Fig. 7B). TLR4 in the HFD group slightly increased, with little difference among five groups. The results of HE staining showed that the hippocampus of the brain in each group showed no obvious difference. The results above showed that inflammatory damage in brains of obese mice was unremarkable. Although the severity of cerebral inflammation was low, there were changes in permeability of the brain. Claudin-1, ZO-1 and occludin of brains in the HFD group were significantly downregulated than the control group. The expressions of claudin-1 and occludin were significantly increased in the Metformin, QWS, QNS group. (Fig. 7C, G–I).

Altogether, the results showed a highly similar trend of variation in colon and brain of TGR5, GLP-1, and tight junctional proteins, while inflammation within the brain tissues was not evident. A comparison of the results disclosed in addition to the colon, quinoa could also regulate the expression of these indicators in brain, and quinoa with saponin performed slightly better than quinoa without saponin.

### Integrative analysis of microbiota.

**FIG 8:**
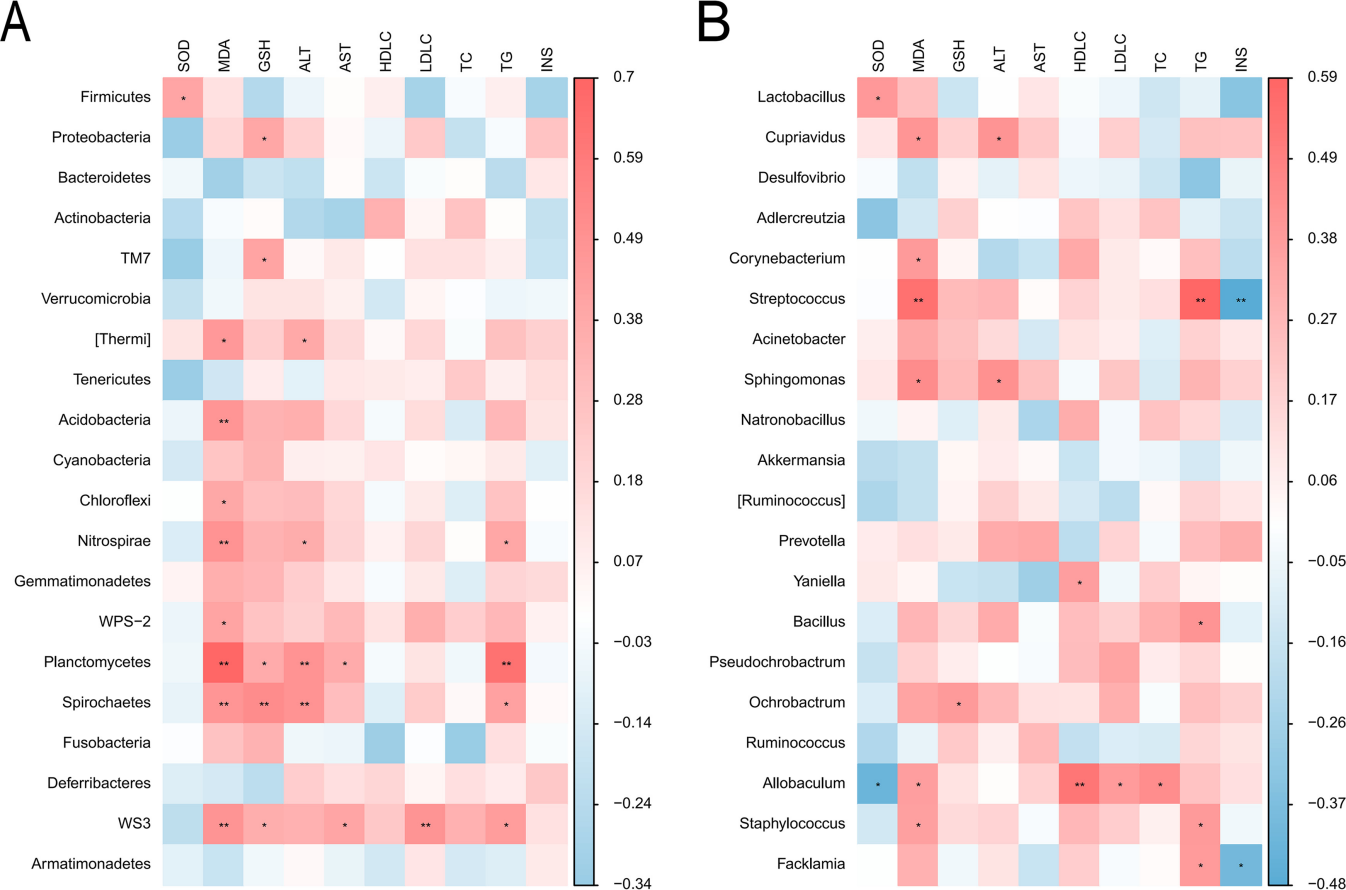
(A) Correlation analysis between the abundance of gut microbiota at the phylum level and the blood indices. (B) Correlation analysis between the abundance of gut microbiota at the genus level and the blood indices. *, *P*<0.05; **, *P*<0.01.

To further confirm the role of microbiota in the obesity and intervention mechanism of quinoa on obesity, we performed the correlation analysis of gut microbiota and the blood indices of obese mice. We selected top 20 relatively abundance of bacteria at phylum and genus levels in colon separately. The results were showed in Fig. 8A and B. At the phylum level, the blood indices with a strong correlation with gut microbiota included MDA, GSH, ALT, LDL-C, and TG. Specifically, MDA had positive correlations (*P*<0.01) with Acidobacteria, Nitrospirae, Planctomycetes, Spirochaetes, and WS3. We also found positive correlations (*P*<0.01) between GSH and Spirochaetes, LDL-C and WS3, TG and Planctomycetes. Meanwhile ALT had positive correlations with Planctomycetes and Spirochaetes (*P*<0.01). At the genus level, MDA had positive associations with Cupriavidus, Corynebacterium, Streptococcus, Sphingomonas, Allobaculum, and Staphylococcus. Among these, the correlation between MDA and Streptococcus was more significant. TG was also strongly related to Streptococcus, but insulin was inversely correlated with it. Moreover, HDL-C had a strong positive relationship with Allobaculum.

## DISCUSSION

Obesity is a complicated disorder which is postulated to be mainly caused by the interactions of environmental triggers and developmental factors. Substantial evidence suggests that disturbed gut microbiota participates in diet-induced obesity and related metabolic disorders (3, 13). Several studies, including our study indicate that quinoa plays an active regulatory role in metabolic diseases including obesity (14, 15). In this study, we focus primarily on the quinoa effects on microbiota in HFD obesity and pathways through which the quinoa in cooperation with the microbiota modulate the obesity. The experimental results suggested that the effects of the quinoa on obesity may be achieved by adjusting microbial composition and microbiota-gut-brain-liver interaction pathway.

In the present study, we constructed a HFD obesity model in mice. Quinoa significantly alleviated the growth rate of weight gain and body fat of obese mice, and the efficacy of metformin and the quinoa with saponin was similar. As obesity normally correlates with declining insulin sensitivity and increased glucose and insulin in blood (16), in addition aberrant activation of hepatic lipogenesis has been implicated in obesity (17), indicators such as the fasting blood glucose and insulin, TC, TG, LDL-C are important predictors of severity of obesity. For these clinical indicators closely linked with obesity, glucose, and lipid metabolism, quinoa displayed a degree of regulatory effects, especially TG, LDL-C. The above results suggest that quinoa has beneficial effects on obesity treatment. The role of quinoa in treatment of obesity has been established, while its mechanisms of action may involve regulations of lipid metabolism, inflammation, and gut microbiota (4, 15). To further explore the treatment mechanism, our study focused on the microbiota-gut-brain-liver axis.

TGR5, also known as G-protein coupled BA receptor (GPBAR1), is an important receptor for bile acids interacting with intestinal flora to alter bile acid composition and regulate signal transduction (18). It has been proven that TGR5 can be activated by BAs and affect glucose metabolism by inducing GLP-1 production. One of the key reasons is that Bacteroides in intestinal bacteria may activate TGR5 and stimulate GLP-1 secretion, then improve glucose metabolism (19). Except glucose metabolism, TGR5 may increase energy expenditure and basal metabolism due to its enhancement of intracellular cyclic AMP (cAMP) (20, 21). In addition, the activation of TGR5 can inhibit LPS-induced upregulation of TLR4-NF-κB pathway, which means the positive role of TGR5 in the suppression of the inflammation to Gram-negative bacteria that release LPS (21, 22). Quercetin and oleanane, the components of quinoa, may be involved in TGR5 upregulation directly or influence TGR5 indirectly through regulation of gut microbiota (23, 24). In our study, the level of TGR5 in obese mice decreased and quinoa with saponin could upregulate the expression of TGR5 in the colon, while quinoa without saponin did not appear to function in this aspect. For gut microbiome, quinoa promoted the relative abundance of Bacteroides and repressed the level of Gram-negative bacteria, especially Enterobacteriaceae, which indicated that quinoa could stimulate TGR5 signaling and process anti-inflammatory effects via modulation of intestinal microbiota. Notably, one recent study reveals that TGR5 is also expressed in hypothalamus, which can protect from the onset and worsening of HFD obesity by activation of the sympathetic nervous system (12). Our experimental results revealed that quinoa with saponin could increase the level of TGR5 in brains, which were in line with our observations in the colon. Quinoa without saponin had the same effect on TGR5 in brains, but the effect was little weaker than quinoa with saponin. We could speculate that quinoa may exert anti-obesity effects through the stimulation of TGR5 to promote glucose and energy expenditure. The activation of the quinoa to TGR5 could be direct or indirect, possibly involving regulation of gut microbiome, and quercetin and oleanane of quinoa might play positive roles.

GLP-1, mainly produced in gut endocrine cells and in the brain, is a peptide that involves in regulating gut motility, satiety, and glucose metabolism (25). GLP-1 is released after food intake and acts to amplify glucose-dependent insulin secretion, and it has been in the spotlight as a target for the treatment of obesity and diabetes as its positive effects on glucose homeostasis, appetite regulation, gastric emptying and postprandial lipid metabolism (26, 27). In addition to TGR5, short-chain fatty acids (SCFAs), metabolites of intestinal microbiota, can promote GLP-1 secretion from L cells and improve glucose tolerance (28). Among them, butyrate is a major metabolite that can stimulates secretion of GLP-1 to augment the insulin, while probiotics, represented by Akkermansia, increase circulating butyrate (29). Additional studies demonstrate that the level of GLP-1 is inversely associated with the abundance of Firmicutes and Bacteroidetes, which may be another evidence of microbiome effects on GLP-1 (30). In our study, quinoa could enable the lowly expressed GLP-1 in HFD group to revert. It has been established that polyphenol-polysaccharide conjugates from quinoa have great potential to stimulate GLP-1 release (31). Meanwhile, this study showed that quinoa with saponin appeared to work better on downregulation of the abundance of Firmicutes and Bacteroidetes and upregulation the abundance of Akkermansia than quinoa without saponin. Thus, besides direct effects on GLP-1, quinoa with saponin may play a key role on GLP-1 via regulation of TGR5 and gut microbiome. GLP-1 is also expressed in the liver and can function as an important factor in the gut-liver axis (32). Besides, GLP-1 exists in the brain and the sources of GLP-1 in brains may be circulating GLP-1 or GLP-1 produced in the brain or peripheral GLP-1 via vagal afferents. And GLP-1 in brains has a neuroprotective action and effects on appetite (33). Quinoa also can promote the expressions of GLP-1 in the liver and brain. Therefore, we speculate that quinoa acts weight loss through the upregulation of GLP-1 in the colon, liver and brain, while quinoa with saponin may had better functions.

TLR4, a receptor of the innate immune system, can be activated by the action of LPS and long-chain saturated fatty acids. TLR4 has been reported to induce systemic metabolic inflammation, ER stress and oxidative stress as its wide distribution in different tissues (34). When it comes to increased leakage of LPS and fatty acids and overloading of lipid, TLR4 and ER stress may be triggered to activate systemic inflammation via cross-interaction, which leads to obesity-associated insulin resistance and oxidative stress (OS) (34–36). The inflammation may induce intestinal epithelial damage and inflict liver injury through the portal vein system (37, 38). It is noteworthy that quinoa has antioxidant and anti-inflammatory effects. Saponins and polyphenols extracted from quinoa can downregulate various cytokines and prevent obesity-induced inflammation (39). Gallic acid and quercetin possess antioxidant activity and improve hepatic inflammation (24, 40). 11S and 2S albumin extract from quinoa exert immunomodulatory effects on macrophage population and may act as TLR4 agonists (41). Quinoa showed distinct downregulation on TLR4 in the colon and liver as well as ER stress, which suggested quinoa could ameliorate inflammatory status in obesity. OS is an important signal to change the homeostasis or maladjustment of ER (42). Regulatory effects of quinoa on SOD and MDA indicated its antioxidant activity. More importantly, quinoa performed significant amelioration of inflammatory infiltrates and hepatic steatosis in the liver, which were consistent with changes of AST and ALT. Simultaneously, quinoa improved the damaged colonic mucosal structure, which might be achieved by elevating the expression of tight junctional proteins in the colon. We surmise that anti-inflammatory effects of quinoa may act directly on tissues, then improve the systemic inflammatory response and insulin resistance. For the overall effect, quinoa with saponin obtained good results. Although brains in HFD group were without obvious signs of inflammation, the levels of tight junctional proteins in brains were significantly reduced, which indicated the disruption of blood-brain barrier (BBB) permeability integrity. Quinoa provided a potent BBB protective effect by increasing tight junctional proteins. It has been shown that the butyrate in SCFAs can attenuate BBB disruption (43). Based on the aforementioned results, we speculate that quinoa may also protect BBB by increasing the abundance of Akkermansia and thus the content of butyrate.

The gut, brain, and liver have intimate interactions, but related studies mainly concentrate on signaling in the nervous system. Intestinal satiety signals can activate hypothalamic lipid-sensitive signals via vagal afferent nerves, which in turn control food intake (44). Intestinal signals transmitted to the brain can also inhibit hepatic glucose production, suppressing obesity onset (45). In addition, the liver can affect hepatic glucose output through the insulin signaling pathway, reducing brain glucose uptake and impairing neuronal cell activity (46). Bidirectional interactions between the gut and liver might occur through the biliary tract, portal vein and systemic circulation. However, recent studies showed that disturbed gut microbiota can disrupt intestinal mucosal permeability and lead to intestinal inflammation, which can lead to liver damage through the enterohepatic circulation, and its metabolites can also affect neuronal cells and synaptic function through the BBB (47–49). Based on our results in the colon, liver and brain, we speculate that quinoa plays an anti-obesity role through gut microbiota acting as an important mediator of the microbiota-gut-brain-liver axis that is regulated by TGR5, GLP-1, and TLR4.

Numerous studies reveal that an increased Firmicutes/Bacteroidetes (F/B) ratio at the phylum level and abundance of Blautia at the genus level are important features of the gut microbiota in obesity, meanwhile diversity and abundance of intestinal microbiota reduce in obese mice (11, 50, 51). Our work demonstrated the foregoing and observed that quinoa could decrease the *F*/*B* ratio and the abundance of Blautia thereby reducing the obesity. Ugural A’s study showed that quinoa may increase the bacterial microbiota, as well as the abundance of Bifidobacterium, Atopobium, Lactobacillus, Prevotellaceae, and so on (52). The soluble polysaccharide fraction from quinoa could reduce the ratio of *F*/*B* and the relative abundance of Proteobacteria in HFD rats (53). The saponin-rich extracts from quinoa may have an antimicrobial action, mainly for lactic acid bacteria and Lactobacillus (54). In the present study, the results indicated quinoa and metformin have similar effects on gut microbiota, were predominantly regulation on Bacteroidetes, Actinobacteria, and Desulfovibrio. The caveat here is that, as a microbial-associated disease without specific pathogen, obesity may be more directly relevant to the dysbiosis of the bacterial ecosystem rather than variations on a particular bacterium (50, 55). Based on our findings, quinoa could make the abnormal intestinal gut flora of obese mice tend toward normalize via an overall role in regulation, with slightly better performance for quinoa with saponin than quinoa without saponin. In addition, quinoa may have indirect influences on MDA, GSH, ALT, LDL-C, HDL-C, and TG via the regulation of abundance of Acidobacteria, Nitrospirae, Planctomycetes, Spirochaetes, WS3, and Streptococcus, Allobaculum in the colon.

## CONCLUSION

In summary, our study proved that quinoa especially quinoa with saponin significantly improved high-fat diet-induced obesity, alleviated the abnormal glycolipid metabolism and reversed gut microbiota dysbiosis, including, but not limited to, the diversity of intestinal microbiota, the ratio of *F*/*B*, relative abundances of Blautia, and functional profiling of microbial communities, especially lipid metabolism, and carbohydrate metabolism. The results of the HFD group in our study showed a canonical microbiota-gut-liver-brain interaction disease model. It is worth noting that, for obese mice the results of TGR5, GLP-1, and TLR4 in the colon are notably similar to those in the brain, which indicates that TGR5, GLP-1, and TLR4 may be essential components of the microbiota-gut-liver-brain axis. At the same time, through microbiota-gut-liver-brain axis, quinoa could upregulate the expression of TGR5 and GLP-1, while downregulate the level of TLR4. Quinoa could also relieve intestinal and cerebral barrier damage as well as abnormally high expressions of ER stress and oxidative stress markers. Future studies will investigate more pathogenesis of obesity and microbiota in different tissues of obese mice, also the regulatory roles of quinoa.

## MATERIALS AND METHODS

### Animals and diets.

All animal experiments were approved by the Institutional Animal Care and Use Committee at Beijing University of Chinese Medicine (BUCM) and conformed to the current existing animal welfare guidelines. The experimental protocols applied in this study were performed in accordance with approved guidelines. C57BL/6 male mice were purchased from Beijing Vital River Laboratory Animal Technology Limited Company (Beijing, China). All mice were adaptively fed for 3 days separately in a standard animal feeding room in the Animal Facility of BUCM before the experiments commenced (room temperature: 23 ± 1°C; relative humidity: 40% ± 10%; light condition: a 12h/12h dark/light cycle). Mice were fed an ordinary diet (crude fiber [≤5.0%], crude ash [≤8.0], calcium [1. 0–1.8%], and phosphorus [0.6–1.2%]) or high-fat diet (24% protein, 24% fat, 41% carbohydrates, fat energy supply ratio was 45%). The diet was purchased from Beijing HFK Bioscience Co., LTD (Beijing, China).

### Obesity induction and intervention procedure.

A total of 30 mice were fed with ordinary diet for 4 weeks, then randomly divided into the control group (6 mice) and the obesity group (24 mice) fed with ordinary and high-fat diet respectively for 8 weeks. The obesity mouse model was identified successful if body weight was 20% greater in HFD mice than the control mice. After high-fat diet intervention to induce obesity, mice from the obesity group were randomly divided into the high-fat diet-induced obesity group (the HFD group), positive control group (the Metformin group), the Quinoa with saponin group (the QWS group) and Quinoa without saponin group (the QNS group) with 6 mice in each group. Mice from the control group were fed with ordinary diet, while mice in the HFD group were fed with high-fat diet. The Metformin group were fed with high-fat diet and received treatment of metformin once a day by oral gavage (1 g/100 g). The QWS group and QNS group were fed with quinoa with saponin and quinoa without saponin (2 g for each mice per day) combined with high-fat diet, and quinoa with saponin and quinoa without saponin were provided by Shanxi Hao Chen Biological Technology Co., Ltd. Intervention lasted for 6 weeks. Body weight and blood glucose were measured at the end of each week and body fat was detected by small animal body composition analyzer at the end of first and sixth week respectively.

### Biochemical indexes evaluation.

The eyeball of mice was picked to draw blood for further detection. The blood was stored in evacuated tubes for centrifugation after 2 h stewing at room temperature to take the upper serum supernatant. Levels of blood glucose (GLU), triglyceride (TG), cholesterol (TC), high-density lipoprotein cholesterol(HDL-C) and low-density lipoprotein cholesterol (LDL-C) were measured by an automatic biochemical analyzer (Hitcha, Japan). Insulin level was detected by radioimmunoassay.

### Hematoxylin-Eosin (HE) staining.

The colon, liver and brain tissues of mice were selected from each mouse and fixed in formalin for 24 h, after which gradient dehydration was performed and tissues were embedded in paraffin for sectioning. The sections were subsequently stained with hematoxylin and eosin and mounted in resin on slides. Images were subsequently captured to evaluate the pathological changes.

### IHC and Western Blotting analysis.

Colons, livers, and brains were fixed in 10% neutral buffered formalin (Beijing Yi-li Fine Chemical, Beijing, China), embedded in paraffin, sectioned at 4–5μm by rotary microtome. IHC for GLP-1, claudin-1 in colons and brains as well as eIF-2α in livers were detected using the rabbit anti-GLP-1 (1:500; Servicebio), mouse anti-claudin-1 (1:500; Servicebio) and rabbit anti-eIF-2α (1:200; Servicebio) monoclonal antibodies. The tissues were examined by laboratory microscopy (Olympus, U-LH100-3 Tokyo, Japan). And the average optical density (AOD) of the positive immunohistochemical reaction was obtained by Image J (1. 53f) for morphometric analysis (the results were presented as μm). The expression of the TGR5, TLR4, GLP-1, occludin, zo-1, CHOP and eIF-2α proteins in colon, liver and brain were detected by Western blotting techniques. The colon, liver, and brain tissues were homogenized, and the protein-containing supernatant was separated and collected. Protein concentrations were determined using a bicinchoninic acid (BCA) kit (Termo Fisher Scientifc, Waltham, USA). Proteins were separated on 10% SDS-PAGE gels and then transferred onto polyvinylidene difluoride membranes. After blocking with 5% defatted milk for 2h, the membranes were incubated with the primary antibodies rabbit GPBAR1 (1:1, 000; ABclonal), rabbit TLR4 (1:1, 000; ABclonal), rabbit anti-GLP-1 (1:1, 000; Servicebio), rabbit anti-occludin (1:1, 000; Servicebio), rabbit anti-zo-1 (1:1, 000; Servicebio), rabbit anti-CHOP (1:500; Proteintech), rabbit anti-eIF-2α (1:500; Proteintech), mouse anti-β-actin (1:5, 000; Biorigin) overnight at 4°C. The β-actin protein was a loading control. Then the membranes were incubated with the appropriate HRP-conjugated secondary antibodies. The bands were visualized with an chemiluminescence reagent (Proteintech, Rosemont, USA) and subsequently scanned and analyzed by Image J (1. 53f). The intensity of the protein bands was normalized to β-actin. All WB experiments consisted of three samples per group.

### Gut microbiota analysis.

Mice were sacrificed and the cecal contents were collected carefully. All of operating procedures strictly followed aseptic principles and all of apparatuses were sterilized by autoclaving. Sterile containers were used to collect samples for 16s rRNA detection. Sequencing of the 16S rRNA gene was performed on an Illumina MiSeq, and data obtained with Illumina MiSeq amplicon sequencing was analyzed with QIIME 1. 8. 0 for the microbiota profiles. Sequences with ≥ 97% similarity were assigned to the same Operational Taxonomic Unit (OTU) by the UCLUST algorithm in QIIME. The representative sequences of each OTU were used for taxonomic identification and phylogenetic analysis. The Chao1, ACE, Shannon and Simpson indices were measured for alpha-diversity. Principal-component analysis (PCA) was used to visualize group separation and identify variations of microbiome data in different groups. Metastats analysis was used to perform contrasts of microbial communities between groups. PICRUSt was used to generate predicted KEGG annotations.

### Statistical analysis.

The data were expressed as means ± standard deviation. One-way analysis of variance (ANOVA), non-parametric test, Dunnett’s T3 were used to analyze the comparisons between groups. GraphPad Prism 8. 0 was used to draw statistical graphs. A *P*-value < 0.05 was considered statistically significant. A repeated measurement process of general linear model was used to conduct one-way ANOVA analysis for repeated measured data (body weight, body fat and fasting blood glucose), and multivariate analysis process of variance was used to make comparisons between groups on each time point. The correlations were identified by Spearman’s correlation, while the correlations analysis was performed by the genescloud tools, and heatmaps were plotted using heatmap tools in the genescloud platform (https://www.genescloud.cn).

### Data availability.

All the relevant data the support our findings are available from the authors on reasonable request. Some have already been included in the paper and the supplemental material. Raw sequence data of microbiota that support the findings in our study have been deposited into NCBI’s Sequence Read Archive under accession number PRJNA832886.
